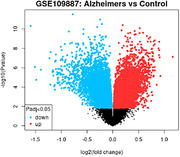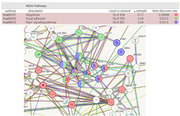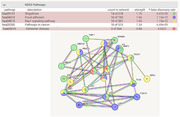# Unraveling Pathways in Alzheimer’s Disease: Insights into Rap1 Signaling Associations with Focal Adhesion and Shigellosis

**DOI:** 10.1002/alz.094760

**Published:** 2025-01-09

**Authors:** Meher Garg, Inhan Lee

**Affiliations:** ^1^ SIU School of Medicine, Physican Pipeline Program, Springfield, IL USA; ^2^ MirCore, Ann Arbor, MI USA

## Abstract

**Background:**

Existing therapeutic approaches in Alzheimer’s disease (AD) targeting beta‐amyloid and tau proteins have shown limited success. Shigellosis, an intestinal infection caused by Shigella, is capable of colonizing the human intestinal epithelium and has been associated with focal adhesions. Rap1 signaling is associated with cancer and cell adhesions. Upregulation of the RAP1 signaling pathway and its interactions with other signaling pathways could contribute to synaptic dysfunction, neuronal damage, and cognitive deficits and has emerged as a candidate pathway for study in Alzheimer’s disease. Associations between the Rap1 signaling pathway, focal adhesion and shigellosis could shed further light on pathogenesis of AD.

**Method:**

We leveraged the Gene Expression Omnibus (GEO) GSE109887 dataset, available on the NIH’s GEO2R website, comprising microarray RNA expression data from medial temporal gyrus of 46 AD patients and 32 controls. Differential gene expression analysis was conducted using GEO2R. Gene interaction analysis and identification of Kyoto Encyclopedia of Genes and Genomes (KEGG) pathways were performed using STRING‐DB. Gene function insights were obtained from Gene Cards. Statistical analysis and heatmap visualization were conducted using R/R Studio.

**Results:**

After analyzing using STRING, the top 250 expressed genes in AD patients were associated with upregulated KEGG pathways. The Rap1 signaling pathway had a false discovery rate (FDR), p‐value of 0.0212, strength of 0.64, and count in network of 10/201. The shigellosis pathway had a FDR of 0.00088, strength of 0.71, and count in the network of 13/218. The focal adhesion pathway had a FDR of 0.0212, strength of 0.65, and count in network of 10/195. The upregulated genes present in Rap 1 signaling and shigellosis were ITGB1, TLN2, PLCB1, PLCE1, and PLCG1. The genes found in Rap 1 signaling and focal adhesion were ITGB1, TLN2, RAF1, PDGFRB, and PRKCG.

**Conclusion:**

Our findings provide valuable insights into the molecular mechanisms underlying AD. The dysregulated pathways in AD show an association between Rap1 signaling and focal adhesion and shigellosis. Upregulation of the Rap1 signaling, focal adhesion and shigellosis pathways could be a biomarker/potential therapeutic target and should be evaluated in future study.